# Theory and Measurement of Heat Transport in Solids: How Rigidity and Spectral Properties Govern Behavior

**DOI:** 10.3390/ma17184469

**Published:** 2024-09-11

**Authors:** Anne M. Hofmeister

**Affiliations:** Department of Earth, Environmental, and Planetary Sciences, Washington University, St. Louis, MO 63130, USA; hofmeist@wustl.edu; Tel.: +1-314-935-7440; Fax: +1-314-935-7361

**Keywords:** heat, diffusion, length-scale physics, absolute methods, dimensional analysis, radiative transfer, thermal expansivity, Young’s modulus, pressure–volume work, mechanisms

## Abstract

Models of heat transport in solids, being based on idealized elastic collisions of gas molecules, are flawed because heat and mass diffuse independently in solids but together in gas. To better understand heat transfer, an analytical, theoretical approach is combined with data from laser flash analysis, which is the most accurate method available. Dimensional analysis of Fourier’s heat equation shows that thermal diffusivity (*D*) depends on length-scale, which has been confirmed experimentally for metallic, semiconducting, and electrically insulating solids. A radiative diffusion model reproduces measured thermal conductivity (*K* = *Dρc_P_* = *D* × density × specific heat) for thick solids from ~0 to >1200 K using idealized spectra represented by 2–4 parameters. Heat diffusion at laboratory temperatures (conduction) proceeds by absorption and re-emission of infrared light, which explains why heat flows into, through, and out of a material. Because heat added to matter performs work, thermal expansivity is proportional to *ρc_P_*/Young’s modulus (i.e., rigidity or strength), which is confirmed experimentally over wide temperature ranges. Greater uptake of applied heat (e.g., *c_P_* generally increasing with *T* or at certain phase transitions) reduces the amount of heat that can flow through the solid, but because *K* = *Dρc_P_*, the rate (*D*) must decrease to compensate. Laser flash analysis data confirm this proposal. Transport properties thus depend on heat uptake, which is controlled by the interaction of light with the material under the conditions of interest. This new finding supports a radiative diffusion mechanism for heat transport and explains behavior from ~0 K to above melting.

## 1. Introduction

To probe the thermal state and thermal evolution of a system requires delineating the behavior of the transport properties of thermal conductivity (*K*) or thermal diffusivity (*D*) of its constituents: These parameters govern heat flow per Fourier’s macroscopic model [1]. The simplest depiction of the model, in one dimension, is as follows:

(1) $$ℑ=-K\frac{\partial T}{\partial z}=-\rho c_{P}D\frac{\partial T}{\partial z}\;\mathrm{and}\;\frac{\partial T}{\partial t}=D\frac{\partial^{2}T}{\partial z^{2}},$$

where *ℑ* is flux, *T* is temperature, *z* is distance, *t* is time, *ρ* is density, and *c_P_* is specific heat at constant pressure *P* (per mass basis). Either transport property suffices, because these are simply linked by the generally well-known properties of *ρ* and *c_P_*, which are static:

(2) $$K=D\rho c_{P}\equiv DC.$$

Heat capacity on a per volume basis (*C*), also denoted as storativity, plays a crucial role in heat transfer per Equations (1) and (2).

Despite the long-standing acceptance of Fourier’s model, along with its simplicity and widespread use in science and engineering, misunderstandings of the physical process of heat transport remain. This is evident in the disagreement of the Wiedemann–Franz law with measured *K* of metals and alloys. However, these data are accurate, as metals make good thermal contacts and are opaque at the thicknesses used (e.g., data and figures in [2,3,4]). Diffusion of heat is a dynamic process, i.e., time is involved, which greatly complicates both theoretical analysis and the extraction of transport properties from experiments.

Misunderstandings of heat transport can be traced to historical developments in classical physics. Specifically, the laws and framework of thermodynamics were advanced circa 1850 without considering the ubiquitous flow of heat, even though Fourier’s model was developed in the early 1800s. This omission is problematic [5] and has contributed misunderstandings or inconsistencies, even to modern studies:

- The kinetic theory of gas connects the motions of gas molecules to temperature by assuming elastic collisions. Because energy is conserved, the system is isothermal, under which condition heat cannot flow per Equation (1).
- Fourier’s model is macroscopic and, therefore, holds irrespective of the microscopic mechanism by which heat moves. This premise has been ignored in commonplace attributions of Equation (1) to describing “conduction”. Actually, his model describes “diffusion” of heat, which is evident in the naming of the governing physical property (*D*) of Fourier’s original formula (Equation (1), right-hand side (RHS)). Moreover, Fick [6] based his model for mass diffusion on Fourier’s formulation.
- Partly as a consequence of the above, diffusion of radiation has been considered a different process than “conduction”. This misunderstanding remains because the term “radiative transfer” lumps two distinct processes together: Under optically thin conditions, as commonly occur at high frequencies, radiation can cross a medium with negligible interaction. This process is denoted as ballistic or boundary-to- boundary transport and is not diffusive, where the medium is essential. Different equations pertain.
- Distinguishing radiative diffusion from heat diffusion (conduction in particular) is a historic remnant of distinguishing visible light from the caloric, the spectral region of which involves infrared frequencies. This distinction, believed by Maxwell, was debated until 1890 [7].
- Transport properties are dynamic, since dimensional analysis of Equation (1)’s RHS shows that these depend on length-scale and time [8] (see below).

Appendix A further discusses modern phonon and electron scattering models for solids and their links to the historic kinetic theory of gas.

Measuring heat transport properties is simple in principle but difficult in practice. Inaccurate data have furthered misunderstandings. Key problems in measuring solids, particularly electrical insulators, include the following:

- Two systematic errors exist with opposite effects: physical contacts provide thermal losses, which artificially reduce *K* (or *D*), whereas ballistic radiative transport artificially enhances *K* (or *D*), such that the amount depends strongly on sample transparency and temperature (e.g., [9,10]). Electrical insulators and semiconductors are affected to varying degrees, depending on the technique.
- Absolute methods solve Fourier’s laws and are inherently more accurate than comparative methods, which rest on using a suitable standard, although some aspects of Fourier’s model may be incorporated [11]. For absolute methods, calibration is not needed, although cross-checks are carried out.
- The length over which heat diffuses must be independently known for a method to be absolute. This has apparently been overlooked during recent experimental developments, which utilize powerful modern computers. Problems are evident in Zhao et al.’s [12] statement that measuring *K* within 5% is difficult even for modern methods. Zhao et al. [12] omitted the highly accurate method of laser flash analysis [13] (±2–3%; see Section 1.1), since Parker et al. [13] developed LFA in 1961.

Appendix B provides more information on conventional contact methods and modern techniques, which are geared to measure thin films and surface coatings.

### 1.1. Synopsis of Laser Flash Analysis (LFA) and Its Advantages

**Figure 1:**
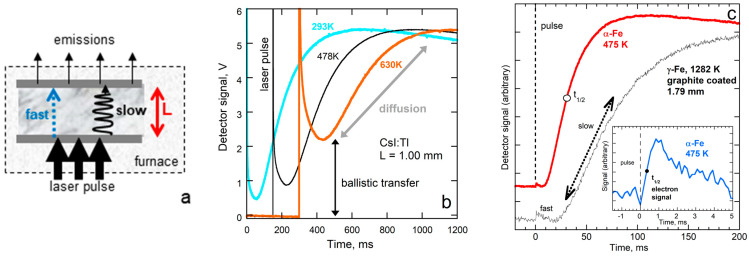
Essentials of laser flash analysis: (**a**) Schematic of the experimental configuration. Dashed box indicates the furnace enclosing the sample. Marble rectangle depicts the cross-section of the sample with thickness *L* (double arrow), which is coated with graphite (grey layers). Arrows indicate the arrival of laser energy and departure of emissions. Blue dotted arrow shows fast ballistic transfer. Squiggle arrows indicate slow diffusive travel of heat across the sample. (**b**) Examples of raw data (time–temperature curves) for a partially transparent electrical insulator. Ballistic transfer (black double arrow) increases strongly with temperature. (**c**) Raw data for an opaque metal (pure electrolytic iron). Circle indicates the half-time for the main (slow) mechanism at low *T*. After the initial (fast) rise, the signal decreases due to the warmed top surface cooling to the surroundings, and then increases again as the majority of heat applied is slowly transferred across the sample (indicated by the double arrow). Inset shows an expanded view of the rapid rise at low *T*. Panel (**b**) from Xu and Hofmeister [25], Figure 1b, with permissions.

Accuracy in LFA is better than in other techniques by a factor of two [14,15,16,17], for several reasons:

- Physical contacts are avoided (Figure 1a), which inhibit heat flow.
- The distance over which heat flows (thickness: Figure 1a) is measured independently and directly, which is essential to describe diffusion. Not adhering the sample to thermocouples and/or heaters permits attaining high temperatures (~2200 K or more). Measurements are now performed routinely down to 150 K [18,19], and lower *T* is possible.
- Monitoring the thermal evolution of the sample (Figure 1b,c) permits direct solution of Equation (1)’s RHS and high accuracy. The physical principles are embodied in the simplest case of adiabatic conditions [13]:

(3) D=0.138785L2t1/2

where *L* is the sample thickness and *t*_½_ is the time taken for the rear surface to reach half of the maximum temperature. This solution assumes that *D* is constant during the measurement, which is achieved by using a short pulse, so that the sample is warmed by less than a few Kelvin.

- Recording temperature vs. time permits resolving multiple mechanisms if they possess significantly different speeds. For highly transparent electrical insulators, a strongly temperature-dependent initial rise following the laser pulse (Figure 1b) occurs due to visible light very rapidly crossing the sample (boundary-to-boundary transport). Time–temperature curves for metals (Figure 1c) demonstrate that rapid electronic transport operates over a brief period [4,20].
- Unwanted effects of ballistic radiative transfer (Figure 1b) are reduced or removed via sample coatings [21] and/or quantitative models [22,23,24]. The underpinnings of this heat transfer/spectroscopic model are described by Blumm et al. [10].
- Accurate determination of *D* also provides accurate values of *K* via Equation (2), since *c_P_* and *ρ* can be accurately measured.
- Samples are fairly small (6 mm diameter or larger, and ~1 mm thick), and effects of background radiation from the surroundings are removed via baseline corrections.

### 1.2. Purpose and Organization

This paper combines an analytical, theoretical approach with data from the absolute, highly accurate method of laser flash analysis. The goal is to advance understanding of heat flow in solids. The focus is on underlying concepts, consolidating key results, and the effect of temperature, because an identity stipulates the effect of pressure on *K* [26,27]. The cited publications provide details on the theoretical analyses and the large datasets underlying these recent discoveries. One objective is to present these findings more simply, and another is to unite results from several papers. Lastly, recent results for thermodynamic properties [28] are extended to include the effects of phase transitions and the behavior of heat transport properties, and then they are validated.

Section 2 (macroscopic theory) shows how Fourier’s law constrains the length-scale dependence of the heat transport properties, discusses the repercussions of heat performing work, and considers the implications of the Stefan–Boltzmann law. Section 3 presents a microscopic (spectroscopic) model for the diffusion of heat. Section 4 covers the LFA method. Section 5 summarizes previous validations of the formulae described in Section 2 and Section 3, focuses on the length-scale dependence of *D*, and verifies the proposal in Section 2.3.2 that connects *D* conversely with *c_P_*. Section 6 discusses implications and presents conclusions.

## 2. Macroscopic Theory

**Figure 2:**
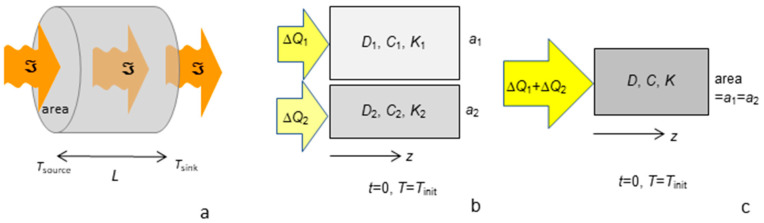
Schematics: (**a**) Longitudinal flow in Cartesian symmetry. At steady state, flux *ℑ* along the special direction is a constant that is independent of position, so the axial thermal gradient is independent of time, and perpendicular slices are isothermal. (**b**) Initial conditions for heat flow along two parallel bars with different areas (*a*) and properties (*D*, *C*, *K*), to which an instantaneous pulse was applied (yellow area). (**c**) Initial conditions for a blended bar, created from superimposing two bars of equal area.

Thermal diffusivity (*D*) and thermal conductivity (*K* ≡ *ρ*c_P_*D*) have been viewed as material properties, as is the case for *c_P_* and *ρ*. Instead, *D* and *K* describe the flow of heat through matter (Figure 2a), as recognized by Fourier, thus depicting how matter interacts with the independent entity of heat-energy over time and space. Neither property pertains to the behavior of unperturbed matter.

Heat flow is ubiquitous. Considering the simplest case of steady-state flow led to different equations for the *T* and *P* responses of several physical properties of solids than those derived classically by considering reversibility and heat (*Q*) storage [28]. The revised formulae and their underpinnings are summarized below.

### 2.1. Fourier’s Law Specifies the Length Dependence of Heat Transport Properties

The dynamical nature of heat transport properties is evident from dimensional analysis of the RHS of Equation (1), which yields

(4) D∝L2τ or D∝uL

where *τ* is a characteristic time and *u* is a characteristic speed, *L*/*τ*.

The LHS of Equation (4) recapitulates Parker et al.’s [13] adiabatic solution (Equation (3)) for one-dimensional flow. Other solutions to Equation (1) have the same form [29]. Length-scale is a crucial variable regarding heat flow in series (Section 2.2.1).

The RHS of Equation (4) shows that thermal diffusivity is the measure of how much any given medium impedes the flow of heat, since speed is a property of the medium and length describes the system. This suggests a linear dependence of *D* on *L* at low *L*. The limit of *D*→0 as *L*→0 is consistent with diffusion requiring a participating medium.

### 2.2. Sum Rules for Slowly Varying Temperature

#### 2.2.1. Series

Steady-state flow of heat across layers that are perpendicular (⊥) to the direction of flow is long understood, since the same applied flux *ℑ* pertains to each interface [30]. If the layer thickness varies,

(5) $$\frac{L}{K_{⊥}}=\frac{L_{j}^{2}C_{j}}{K_{j}}\sum \frac{1}{L_{i}C_{i}}=\sum \frac{L_{i}}{K_{i}}.$$

If the *C_i_* is similar for the layers, then a harmonic mean also holds for *D*.

#### 2.2.2. Parallel Layers or Multiple Mechanisms in a Homogeneous Medium

Previous descriptions of parallel (||) flow as *K*_||_ = Σ*K_i_* originate by analogy to electrical currents [31]. This analogy is inappropriate because heat, unlike charge, flows into, across, and out of any solid (Figure 2a).

Equations for heat flow in parallel bars of equal area (Figure 2b,c) were derived by Criss and Hofmeister [4] from Fourier’s laws by conserving heat energy (the adiabatic approximation). The resulting summation, $K\sum C_{i}\frac{\partial T_{i}}{\partial z_{i}}=C\sum K_{i}\frac{\partial T_{i}}{\partial z_{i}}\;$, was simplified to account for common relationships among the component volumetric heat capacities (*C_i_* = *ρ_i_c_Pi_*) and diffusivities (*D_i_*), as follows:

For *n* independent mechanisms, *C* = Σ*C_i_* in bars of equal area, such that the thermal evolutions associated with the mechanisms are similar:

(6) $$K_{\left|\right|}=n\frac{\sum C_{i}K_{i}}{\sum C_{i}}\;\mathrm{for}\;D_{i}\approx D_{j}\;(\mathrm{independent}\;\mathrm{mechanisms}).$$

Allowing for different cross-sectional areas of the bars modifies Equation (6) to

(7) $$K_{\left|\right|}=\frac{\sum f_{i}C_{i}K_{i}}{\sum C_{i}}=\frac{\sum f_{i}C_{i}K_{i}}{C}\;$$

where the volumetric fractions (*f_i_*) sum to *f* = 1. Essentially equal *C_i_* values provide the arithmetic mean.

For another importance case of one mechanism but with multiple carriers, *C*_single_ = Σ*C_i_*/*n*. This leads to a simpler equation of *K*_single_ = Σ*C_i_K_i_*/Σ*C_i_*. For nearly equal *C_i_*, this reduces to *K*_single_ = <*K_i_*>, as expected. This result confirms that the kinetic theory of gas errs by a factor of three (see Appendix A).

#### 2.2.3. Multiple Mechanisms in Metals and Alloys

When the values of *D_i_* differ, the temperatures associated with the mechanisms evolve at very different rates. Also, allowing *C_i_* to differ [4] gives

(8) $$K_{\left|\right|}=\frac{\sum C_{i}}{\sum C_{i}^{2}}\sum C_{i}K_{i}\;\mathrm{for}\;C_{i}\neq C_{j}.$$

For metals, electronic and lattice mechanisms have much different values for specific heat: *C*_ele_ << *C*_lat_ = *C*_meas_. Because electrons have fast speeds, *D*_ele_ >> *D*_lat_, which reduces Equation (8) to

(9) $$K_{metal}=K_{lat}.$$

Importantly, traveling electrons entering a given region are “hotter” than the surroundings. Because traveling hot electrons cannot take up heat from the colder regions they enter per the second law of thermodynamics, they cool and equilibrate. Unoccupied states exist for the traveling electrons to fall into, because states differ between the hot and cold regions. Hence, electronic heat transport is transient in a metal. See [4,20] for further discussion.

#### 2.2.4. Grainy Media with Similar Grains

Both series and parallel flow occur as heat flows through a grainy medium. Thus, Merriman et al. [32] averaged Equations (5) and (7), while replacing *L_i_*/*L* in Equation (5) with *f_i_*. The simplest possible case, when the heat capacities of the phases are quite similar, is as follows:

(10) D=12∑fiDi+12∑fiDi−1

### 2.3. Thermophysical Behavior of Solids during Incremental Addition of Heat

In a gas, diffusing molecules carry heat with them. In contrast, heat flows through a solid (Figure 2). Classical thermodynamics provides an identical set of equations for both states of matter because rigidity, which distinguishes solids from gases, was neglected. Classical thermodynamics centers on reversible conditions, where entropy *S* ≡ *Q*/*T* depends on the stored heat (*Q*). Instead, during heat flow, the relevant quantities are the flux *ℑ*, which defines the rate at which heat flows past an area, *K* or *D*, and also the boundary and initial conditions.

Heat always flows. Considering realistic and achievable steady-state conditions for elastic solids resulted in several new thermodynamic relationships for solids [28]. Those involving temperature changes are summarized in Section 2.3.1, and a new relationship involving *D* is proposed in Section 2.3.2.

#### 2.3.1. Consequences of Heat Performing Work on Static Properties

Pressure–volume work performed upon adding heat externally (ext) is quantifiable:

(11) $$\;c_{P}M\Delta T=\Delta Q_{\mathrm{ext}}=\mathrm{work}=PdV=F\Delta L,$$

where *F* is the force needed to expand the bonds with average length *L*. Since *P*-*V* work occurs, the relevant physical properties are the equation-of-state parameters of isothermal bulk moduli and isobaric thermal expansivity (*α*). The definitions are as follows:

(12) $$\beta_{T}\equiv -\frac{1}{V}{\left.\frac{\partial V}{\partial P}\right|}_{T}=\frac{1}{\rho }{\left.\frac{\partial \rho }{\partial P}\right|}_{T}=\frac{1}{B_{T}}\;\mathrm{and}\;\alpha_{\mathrm{vol}}\equiv \frac{1}{V}{\left.\frac{\partial V}{\partial T}\right|}_{P}\;$$

The subscript “vol” for volume is used because measurements of expansion are often linear “lin”, and these values for α differ by a factor of three for isotropic matter.

Increasing *V* (or *L*) is opposed by the interatomic bonding forces. The resistance of a solid to changes, i.e., its strength, is embodied in Young’s modulus (Ξ ≡ 9*BG*/(3*B* + *G*), where *G* is the shear modulus). Shear moduli are measured in acoustic or elastic experiments [33]. Bulk moduli are also measured in elastic or acoustic experiments, and they are designated as “adiabatic” but are denoted as isentropic (*B_S_*). This disparity exists because *B_S_* is defined in classical thermodynamics, but *B_Q_* is not. Importantly, within experimental uncertainties, elastic determinations of bulk moduli equal those determined from volume (or length) changes for hundreds of substances at ambient and elevated temperatures [28].

For incremental length changes in solids, *F* is proportional to Ξ × area. Considering a spherical volume around an atom in evaluating Equation (11) gives

(13) $$c_{P}M\approx \Xi 4\pi L^{2}\frac{\Delta L}{\Delta T}=\Xi 4\pi L^{3}\frac{\Delta L}{L\Delta T}=\Xi V\alpha_{\mathrm{vol}};\;\mathrm{more}\;\mathrm{generally},\;\rho c_{P}\propto \alpha_{\mathrm{vol}}\Xi .$$

Using the proportionality on the RHS recognizes that the *F* experienced by any given atom depends on the number of bonds around each atom (i.e., atomic coordination of the structure). For polyatomic and diatomic compounds, the proportionality constant in Equation (13) is related to number of cations (*N*) divided by the number of atoms in the formula unit (*Z*), yielding various ratios of physical properties:

(14) ρcP=αvolΞNZ or cPαvol=ΞNZρ or αvol=ρcPΞNZ.

Equation (14) also describes monatomic solids if instead *N* is the number of cations in the unit cell and *Z* is half the number of nearest neighbors in that unit cell.

#### 2.3.2. Consequences of Heat Doing Work on Dynamic Properties

In static experiments, incremental addition of heat raises the temperature and simultaneously does work. Raising the temperature means that more heat is stored. Heat that is stored is unavailable to flow. However, the definition of *K* = *ρc_P_D* implies that more heat should flow as more heat is stored if *D* is unaffected. Therefore, *D* must decrease as more heat is taken up and stored. We propose the following converse relationship:

**Theorem** **1:** *Thermal diffusivity responds oppositely to specific heat during continuous and discrete changes:*

(15) $$\mathrm{If}\;c_{P}↑\;\mathrm{then}\;D↓\;\mathrm{and}\;\mathrm{conversely}.$$

**Proof:**  Experimental verification is provided in Section 5. □

### 2.4. Implications of the Stefan–Boltzmann Law

Stefan’s [34] measurements of emissions of strongly absorbing materials are essential to understanding heat transport. The modern form for his discovery is as follows:

(16) $$ℑ=\frac{℘}{4\pi s^{2}}=\sigma_{\mathrm{SB}}T^{4}.$$

where ℘ is the power emitted (also known as luminosity, ₤), *s* is the spherical radius, and *σ*_SB_ is the Stefan–Boltzmann constant.

#### 2.4.1. Surface Emissions for All States of Matter Are Described

Although Stefan’s result was initially considered to only describe solids, Equation (16) applies to all states of matter if the body is sufficiently large. Confusion persists because measurements of partially transparent solids are affected by back-reflections of this flux just inside the surface, as shown theoretically and confirmed experimentally [35,36,37].

The Stefan–Boltzmann law is applied to diverse types of stars [38], some of which have gas-like average densities. For a gassy body, surface reflections do not exist. However, colder gas species in stellar atmospheres can reabsorb the surface emissions, while dwarf stars (e.g., the Sun) have a fluid surface, which does reflect. Verification exists in the simple power-law relationship between ₤ from Equation (16) and stellar mass across most of the main sequence (e.g., [39]).

#### 2.4.2. Applicability to Any Geometry and Any Pressure

The middle term of Equation (16) is specific to a sphere, but not the LHS or RHS, and so the Stefan–Boltzmann law is valid for objects other than spheres. However, surfaces of constant *ℑ* must be congruent with the coordinate system.

A key characteristic of energy is that, unlike matter, any amount of energy can occupy a given space. Consequently, flux is unaffected by *P* and depends only on *T*.

#### 2.4.3. Applicability to Object Interiors and Generality

If heat is transferred solely by diffusion (i.e., ballistic transport is negligible), then *ℑ* inside an object is controlled by *T* at that interior position. As *P* is immaterial to *ℑ*, the Stefan–Boltzmann law holds inside a body of any size, no matter how compressed it is by gravity. Temperature is controlled by Fourier’s laws, *K*, and circumstances such as boundary conditions.

Diffusion governing interior heat transfer, coupled with Equation (16), requires that blackbody radiation is the entity diffusing, for several reasons:

- The Stefan–Boltzmann law is mathematically obtained from the blackbody curve via integration over frequency (e.g., [8] (p. 256ff)). Historical spectroscopic experiments on graphitized platinum wires (e.g., [40]) have been taken as proof of Planck’s function describing the frequency (*ν*) dependence of blackbody emissions.
- Because Planck’s function has the same mathematical form for any *T* and covers *ν* from its limits of 0 to ∞, its integrated form (the Stefan–Boltzmann law) holds regardless of the specific temperature or details such as the microscopic mechanism. Equation (16), like Fourier’s model, is a macroscopic model.
- Theoretical analyses of emission spectra from small objects composed of partially transparent materials assume that blackbody radiation is the entity diffusing [35,36,37]. Spectroscopic measurements of variously sized objects with controlled thermal gradients [37] validate this model and its assumptions.
- Similarly, models of electromagnetic (EM) radiation diffusing across a medium [41,42] are based on the blackbody function. This model is widely accepted in astronomy [43]. Although discussions center on the visible region, the integrals used (Section 3) extend from *ν* = 0 to ∞. Independence of spectral properties on *T* over all frequencies of light is required for the popular form of *K*∝*T*^3^ [44], but this stipulation is not met by real materials.

## 3. Microscopic Model for Conduction (Diffusion) of Heat in Solids

As discussed in Section 2.4, the link of the Stefan–Boltzmann law to the blackbody curve shows that EM radiation is the entity propagating during diffusive heat transfer. Additional points relevant to the microscopic nature of heat transfer include:

- EM radiation is pure energy and, unlike phonons or electrons, can enter, traverse, and exit a body (Figure 2a). Furthermore, phonons are pseudo-particles.
- Opacity does not exist, except at surfaces due to back-reflections, because no material completely reflects, absorbs, or transmits at any frequency. Fourier’s laws describe diffusion of heat in the interior, not these edge effects.
- The uptake of heat is macroscopically regulated by *c_P_*, which depends on the absorption of radiation in vibrational transitions, i.e., on the spectra of the material.

Because *K* is measured at laboratory temperatures (mostly below 1000 K, whereby blackbody emissions are intense at low frequencies), thermal conduction results from the diffusion of radiation predominantly in the infrared (IR) region, where solids strongly absorb [8] (pp. 359–398), as follows:

### 3.1. A Spectroscopic Model for K

The basis is the integral equation for diffusion of blackbody radiation (e.g., [41,42,43]). Along a Cartesian direction (or per steradian, if radial), diffusion of electromagnetic energy is governed by

(17) $$K_{rad,dif}(T)=\int_{0}^{\infty }\frac{1}{A(\nu ,T)}\frac{\partial I(\nu ,T)}{\partial T}d\nu ,$$

where *I* is the intensity of the blackbody radiation and *A*(*ν*) is the absorption coefficient of the material. Diffusion requires optically thick conditions, i.e., *AL* > 1 or 2 over the relevant distance (*L*), where *T* changes significantly for all frequencies involved. The integral is not relevant to heat transfer for spectral regions where *AL* < ~1. If *I* is intense over such transparent spectral regions (typically in the near-IR or visible region), ballistic transport occurs. LFA experiments with partially transparent solids use a different model to remove the ballistic overprint on an otherwise diffusive response (see [10,22,23,24], Figure 1, and Section 4).

Physical scattering is not considered in our model. Sum rules (Section 2.2.2) could be applied to *K* calculated from Equation (17) to model inhomogeneous grainy media.

### 3.2. Simple Spectra Yield Analytical Solutions to Diffusion of Radiation

Spectral data over a wide range of *T* and *ν* are rare. However, integration is a smoothing function. Metals have bland spectra (e.g., [45]), so a simple function is reasonable. Insulators have variably strong peaks in the IR region, but these commonly overlap. Because simple functions such as the boxcar provide accurate estimates of heat capacity for insulators with diverse spectral patterns (e.g., [46,47,48]), this should be the case for calculating *K*. Moreover, optically thick conditions in the infrared region occur for *L* << 0.1 mm for insulators, so an average *A* for the infrared retion represents diffusion under laboratory conditions. Metals absorb more strongly, but *A* cannot be infinite.

For proof of concept, and to provide analytical formulae, Equation (17) was evaluated for three cases, using formulae from [8] (pp. 379–391). One important idealization is a boxcar shape for *A* from *ν* = 0 up to some cutoff, which gives

(18) $$K\left(T\right)=const.\left[\frac{24}{b^{3}}-\frac{e^{-b\nu }}{b^{3}}\left(24+24b\nu +12b^{2}\nu^{2}+4b^{3}\nu^{3}+b^{4}\nu^{4}\right)\right];\;b=\frac{1.44}{T}.$$

where *b* is related to Planck’s constant, and *ν* is the cutoff here. The “constant” is related to the maximum *K* of a given material. However, a boxcar has finite *A* at *ν* = 0, but media cannot absorb light when none exists. A triangular approximation to *A*(*ν*), where *A* = 0 at *ν* = 0, meets this stipulation and likewise yields a power series upon integration:

(19) $$K\left(T\right)=const.\left[\frac{6}{b^{2}}-\frac{e^{-b\nu }}{b^{2}}\left(6+6b\nu +3b^{2}\nu^{2}+b^{3}\nu^{3}\right)\right];\;b=\frac{1.44}{T}.$$

Another possibility for a triangular function is *A*~*ν*^2^, so

(20) $$K\left(T\right)=const.\left[\frac{2}{b}-\frac{e^{-b\nu }}{b}\left(2+2b\nu +b^{2}\nu^{2}\right)\right];\;b=\frac{1.44}{T}.$$

Equations (18)–(20) can be summed to represent real, measured spectra, and the results can be fitted to data with different scaling constants and frequency cutoffs.

## 4. Materials and Methods

### 4.1. Laser Flash Analysis

Specimens in our LFA 427 apparatus from Netzsch Gerätebau, Germany, were held in a graphite furnace under an Ar gas atmosphere. For a detailed description of the apparatus, see [49]. The samples were thin slices, usually disks of 10 mm diameter and 1 mm thickness. The surfaces were ground with ~10 to 50 μm diamond pads, striving for parallel faces, and then coated with graphite spray.

Figure 1a provides a schematic of the principles of operation. The temperature dependence of *D* was obtained by varying the furnace temperature, which was measured to within ~1 °C using a calibrated W-Re thermocouple. A laser pulse supplied a small amount of heat to a graphite basal coating, which also buffered the oxygen fugacity. As this increment of heat diffused from the bottom coat to the top coat of the sample, the time dependence of thermal emissions was recorded with an InSb detector (Figure 1b,c). Cowan’s [50] algorithm, which goes beyond Equation (3) to address cooling to the surroundings, was used for samples with negligible ballistic radiative transport. Mehling et al.’s [24] model was used to account for spurious radiative transport that was not removed by coating the sample (Figure 1). See [51] for examples of its application, the relevance of the material absorption coefficients, and additional procedural details.

Room temperature data were also collected using an LFA 467 also from Netzsch with a 0.01 ms pulse from a xenon flash lamp at 250 volts, with 4 to 12 data acquisitions. See Lindeman et al. [19] for details on the instrument. Temperatures were measured using calibrated type-S thermocouples. We used finite pulse corrections [52] and a single sample in each of the 4 possible positions to attain the highest possible accuracy.

Standards were used to verify the accuracy of both instruments. Uncertainties in *D* of ~2 to 3% arose mostly from thickness determinations and faces deviating from parallel.

### 4.2. Samples

Most of the samples are described in the cited literature. Appendix C provides information on the samples used to produce data first presented here.

## 5. Experimental Assessments of the Theory

Except for Equation (15), our macroscopic and microscopic models of Section 2 and Section 3 have been validated previously. A brief summary is presented, with additional data on the length dependence of *D* and a brief discussion of its pressure dependence. We then evaluate how heat transport properties depend on heat uptake.

### 5.1. Demonstration of Length-Scale Physics

**Figure 3:**
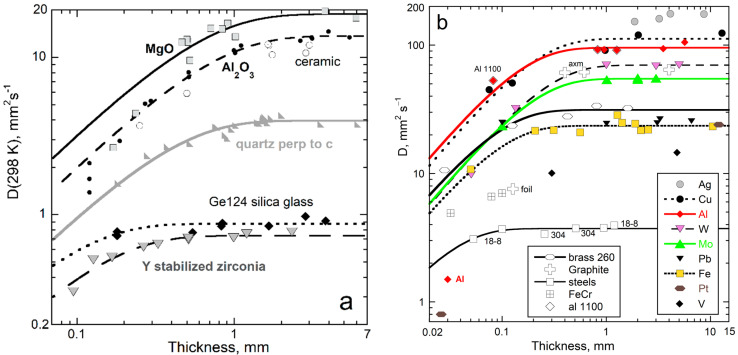
Dependence of *D* at 298 K on thickness. Least squares fits are to *D*(*L*, 298 K) = *D_∞_*[1 − exp(−*bL*)], where the fits have *b* values that roughly inversely correlate with *D_∞_*, representing very large samples: (**a**) Insulators. Symbolts as labelel. Note the rapid drop in D for the thinnest MgO samples (squares), which fall on the trend for single-crystal corundum (dots). (**b**) Graphite, metals, and 3 alloys, where a 0.01 ms pulse was used for *L* < 1 mm. Part (**a**) modified from Figure 7.9 in Hofmeister [8], with permissions. Part (**b**) combines previous results from [26,53] with 5 additional samples; see Appendix C.

Measurements using LFA demonstrate that the heat transport properties of homogeneous solids depend on length (Figure 3). The predicted linear dependence (*D* = speed × *L*) is observed for *L* < 1 mm for insulators, and for *L* < 0.1 mm for many metals and alloys [53]. Similarly, dependence of *D* on *L* is suggested for SiC and diamond, and this was quantified at high *T* for Al_2_O_3_ [8], MgO, and cubic zirconia [26]. Thermal conductivity behaves likewise, per Equation (2).

Dependence on length results from diffusion requiring a medium that interacts with the flowing heat. When scant material exists, heat cannot diffuse and instead crosses ballistically. For the insulators, ballistic transport becomes increasingly strong as *L* decreases below ~1 mm. This contribution was removed from the data per [24], so Figure 3 depicts diffusion only.

The length-scale dependence was not observed earlier for several reasons: Thickness is not always reported, and the ballistic correction to LFA postdates 1998. Other algorithms are too uncertain to resolve spurious radiation, use *L* near 2 mm where *D* is constant, or probe thin films but assume thickness (e.g., thermoluminescence [12]).

### 5.2. Verification of Sum Rules for Parallel Flow

#### 5.2.1. The Two Different Mechanisms in Metals

For metals, transient transport manifests as a tiny rise following the pulse (e.g., Fe: Figure 1c). Rapid rises for stainless Al, V, Ni, Ag, Pb, Co_94_Mo_6_, steel 304, and brass 360 are shown in [4].

Transient electronic transport is expected due to the high speeds of electrons. A weak rise is expected due to low electronic heat capacity. The tiny rise has the following characteristics: It is slower than the fast rises produced by visible radiation crossing an insulator and depends much more weakly on temperature (Figure 1b,c). Other characteristics of the tiny rise indicate electronic transport [4]. In particular, electronic transport is rapidly attenuated due to strong electron–electron interactions [4].

The tiny rise was sufficiently resolved from the laser pulse for 17 different metals and alloys to permit quantitative analysis [4]. The shape of the rise matched that of Cowan’s [50] model, which shows that the tiny rise results from diffusion of heat. Some findings from [4] are highlighted here.

**Figure 4:**
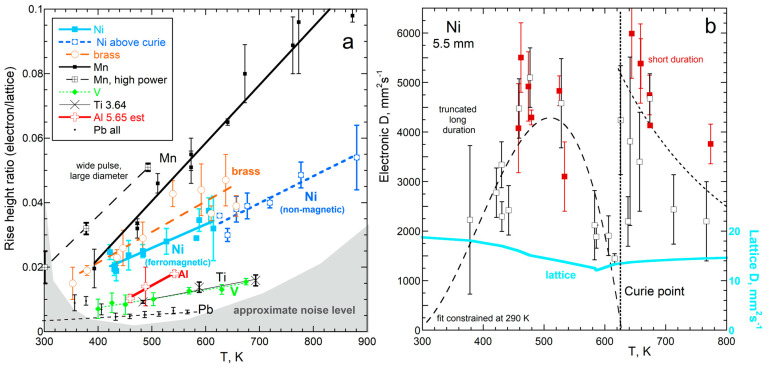
Comparisons of electronic tolattice heat transport in metals: (**a**) Temperature dependence of the ratio of the rapid to the slow rise heights for an assortment of non-magnetic metals, plus nickel below the Curie point. Least squares fits were not forced through the origin. (**b**) Results for Ni acquired in many different runs. Filled squares = short data collection times. Open squares = truncated, long collection times. Above *T*_Curie_, a power-law fit best describes the data. Part (**a**) was reproduced from Figure 11b, whereas part (**b**) was from Figure 13c, both from the work of Criss and Hofmeister [4], which has a Creative Commons 4 license.

The electronic signal intensity is proportional to the lattice signal intensity (Figure 4a) because both carriers are stimulated by the same pulse (Figure 2). This behavior is consistent with assumptions underlying our heat-energy-conserving model (Section 2.2). Conditions for one run on Mn permitted data collection near room temperature. For other samples, the signals were weaker, requiring *T*~473 K, where the detector of the LFA 427 is more sensitive.

Thermal diffusivities for electrons extracted using Cowan’s model are ~200 × *D* of the lattice (Figure 4b), which is consistent with their respective carrier speeds. Figure 4b shows results from the two types of measurements: “long durations” probe both the rapid and slow rises, as shown in Figure 1c and Figure 4a, where the electronic part is analyzed by truncating the signal at small times; “short duration” measurements collected data for a narrow time interval after the pulse to improve the signal-to-noise ratio and better quantify the rapid rise. Differences in *D*_ele_ between short and long collection times above the Curie point (Figure 4b) are attributed to the very high values not being well constrained for the lower-resolution measurements and higher noise at high *T*. At the Curie point, while the transition was occurring, the results for *D*_ele_ varied widely and were not included in fitting.

Electronic thermal diffusivity depends on chemical composition, temperature, phase transitions, cation disorder, and length-scale [4]. Electronic transport is much more strongly affected by the Curie point than lattice transport (Figure 4b). Measurements of Ni and invar indicate that the non-magnetic state has higher *D*_ele_ than the magnetic state. Different responses show that the two mechanisms are independent, which is consistent with the nearly-free electron model.

**Figure 5:**
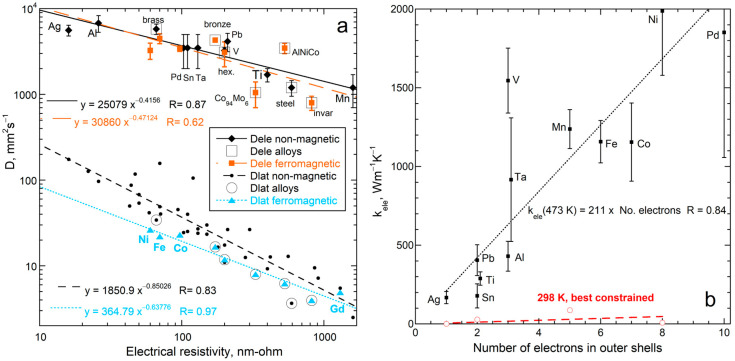
Dependence of metallic heat transport properties on physical properties: (**a**) Plot of *D* vs. electrical resisitivity. Measurements of *D*_ele_ are at 473 K. For *D*_lat_, our data and direct measurements compiled by Touloukian et al. [54] are used, which are uncertain by ~2 and ~5%, respectively. (**b**) Relationship of *k*_ele_ with the number of loosely bound electrons. Ti and Ta were shifted slightly to the right for clarity. Part (**a**) is from Figure 17, and part (**b**) is from Figure 20b, both inform the work of Criss and Hofmeister [4], which has a Creative Commons 4 license.

Commonalities also exist. As discussed in diverse sources, the motions of electrons and phonons are each impeded by interactions with electrons, valence electrons of the vibrating cations, and defects, as well as by magnetism. Hence, the same factors should affect the transport of heat by each carrier, although the specific responses should differ. Figure 5a shows such behavior: both *D*_ele_ and *D*_lat_ depend on resistivity, but with different proportionalities. Differences are evident, with the number of conduction electrons strongly affecting *D*_ele_ (Figure 5b). Lattice *K* instead depends on interatomic distance ([4]; Figures 22 and 23 therein).

#### 5.2.2. Grainy Media with Grains of Similar Storativity

Merriman et al. [32] measured *D* for 60 sections from 28 well-characterized silicate rocks to test our model of Section 2.2.2. The compositions and orientations of the mineral grains in the sections were measured, along with the average grain size, which ranged from 0.008 to 3 mm. Most sections were close to 0.1 mm. The proportions of thmineral phases in the slices and bulk rocks were determined from X-ray diffractometry (XRD) analysis of powders. Rock density, porosity, and bulk chemical composition were also measu measured.

The literature data constrain density and specific heat for the minerals composing these rocks. Our large mineralogical database on *D* was used to evaluate the formulae. We did not evaluate samples that had large proportions of minerals for which *D* is unknown (namely, Fe-rich carbonates, sulfides, serpentine, or barite) or highly porous samples (marbles and some quartzites).

The rock suite is dominated by quartz (SiO_2_) and plagioclase feldspars, which are solid solutions (mixed crystals) of NaAlSi_3_O_8_ and CaAl_2_Si_2_O_8_. Some sections have preferred orientations of either quartz, which has high *D* in one orientation [55], or micas, which have very low *D* across the layers [56]. The plagioclases have low *D* in all three orientations [57].

**Figure 6:**
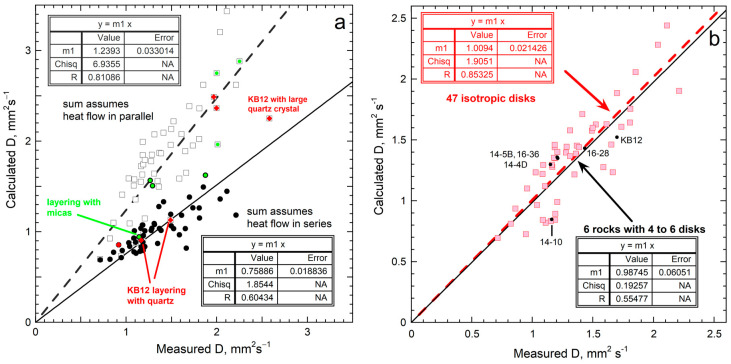
Comparison of thermal diffusivity calculated from XRD mineral proportions to LFA measurements on non-porous silicates: (**a**) Data on individual disks. Disks with special attributes are labeled. Sections with oriented minerals were plotted using the appropriate formula for the orientation. (**b**) Comparison of the average *D* calculated for series and parallel heat flow to *D* data on isotropic sections. Squares = silicates. Dots = averages for 6 rocks for which 4 to 6 disks were measured. Modified from Figures 12b and 14 in the work of Merriman et al. [32], with permissions.

Figure 6a demonstrates that series flow (Equation (5)), with *f* instead of *L*) underestimates *D* for isotropic rocks, whereas parallel flow (Equation (7)) overestimates *D* for isotropic rocks, by roughly equal amounts. For 11 of the 13 layered rocks, using the appropriate formula reasonably reproduces *D* from mineral data.

Figure 6b shows that, within ~5%, isotropic rocks follow Equation (10), which is analogous to the Voigt–Reuss–Hill average for elastic moduli [58]. The thermal conductivity results are similar [32]. Our approach is more certain than the nominal value (±5%) claimed in diverse methods for measuring the heat transport of insulators, but rarely attained ([12], Appendix B).

The spread about the average for isotropic rocks (Figure 6b, squares) is mostly caused by sample inhomogeneity, because scatter is much less about the fit to samples where multiple slices of the same rock were measured, even though few rocks meet this criterion (Figure 6b, dots). Grain size appears to be unimportant, except for section KB12, which had a large grain of high-*D* quartz that spanned the thickness but did not fully cover the area. Uncertainty also exists in the mineral proportions in the disks, and because our database on *D* [8,32] did not contain information on all the phases. A few were estimated from minerals with the same structure but slightly different compositions. Rock 14-10 is more uncertain than the others, as 29% of the sample was a mineral without *D* data.

### 5.3. Verification of Our Radiative Diffusion Model

The thermal conductivity of all materials rises steeply from its limit of zero at absolute zero, reaching a peak between ~20 and 300 K, and then declines gradually to some high temperature (e.g., [59]). Some materials have a weak second peak in *K*(*T*) at higher temperatures. Section 5.3.1 validates our analytical model against *K* below 1200 K.

At high *T*, thermal conductivity and diffusivity behave similarly. For structurally complex insulators, the gradual decline in *D* with *T* reverses, providing a minimum or nearly constant behavior near 1000 K, and likewise for *K* [8,60]. Section 5.3.2 discusses accurate measurements of *D* at high temperatures, showing that these also confirm radiative diffusion.

#### 5.3.1. Thermal Conductivity Measurements at Low Temperatures

The key features of *K* (a cryogenic peak and gradual decline to high *T*), which pertain to thicknesses of a few mm, are reproduced by our two-parameter radiative diffusion model, which assumes a simple function for absorption spectra. One parameter, “const.” in Equations (18)–(20), defines the value of *K* at the peak. The second parameter, cutoff *ν*, defines the peak sharpness. The decline in *K* with *T* results from the blackbody curve underpinning the model, where its specifics are controlled by these two parameters combined.

**Figure 7:**
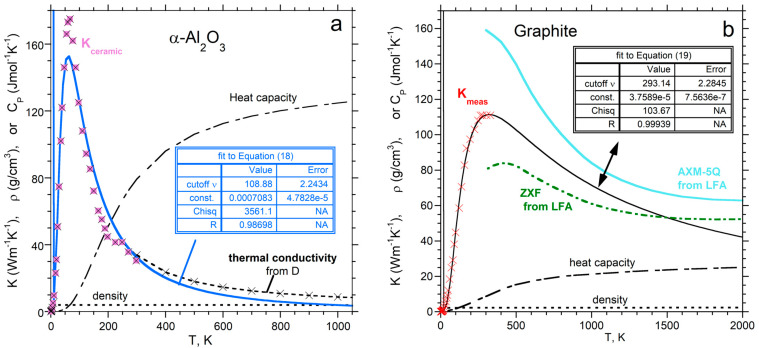
Fits of our 2-parameter radiative transfer model to thermal conductivity data: (**a**) Electrically insulating Al_2_O_3_, represented by a simple boxcar spectrum. Squares = measured *K* on ceramic corundum [64]. Dot-dashed line = *K* obtained from LFA data on sapphire and ceramic Al_2_O_3_ [61], using *c_P_* from [65] and *ρ* from [66]. (**b**) Semiconducting graphite represented by a triangular ramp spectrum. Plus sign = measured *K* from [62]. Low-*T* data on *C_P_* for pyrolytic graphite [67]. Dot-dashed and blue lines = *K* obtained from LFA data, using high *T* density and specific heat from [63]. Modified from Figure 11.5a,b in the work of Hofmeister [8], with permissions.

For insulating corundum, our radiative diffusion model reasonably represents *K*(*T*) from nearly 0 K to ~1000 K (Figure 7a). The fit assumes a simple boxcar for Al_2_O_3_ absorption spectra. The low-*T* data are affected by contact resistance and ballistic transport, given the variable results for different samples in the literature. Peak height is much higher for gem-quality samples. At high *T*, gems and ceramics give nearly identical values for *D* and, thus, *K* [61].

The two-parameter fit to graphite using a ramp (*A* ~ *ν*: Figure 7b) matches the cryogenic measurements reported in [62]. The predicted tail is roughly intermediate between measurements on other graphites. Graphite is porous, with variable grain size and impurities, producing variations in *K* [63].

**Figure 8:**
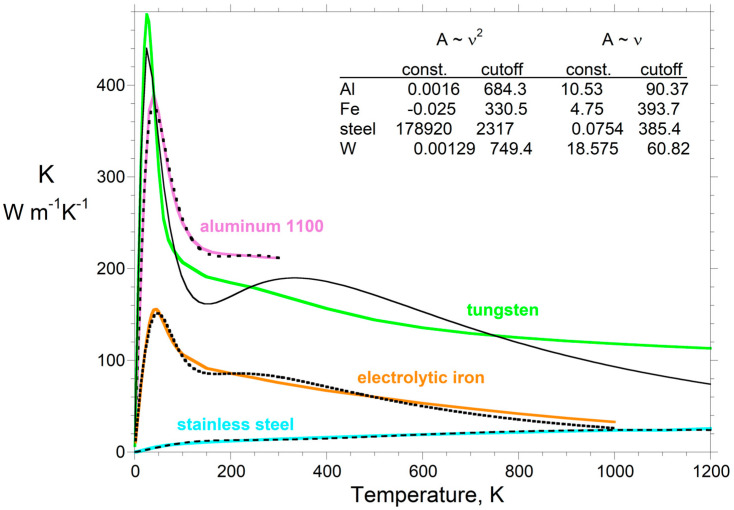
Fits to pure iron, tungsten, and two alloys using a radiative diffusion model that sums Equations (19) and (20). The four parameters are tabulated. Data are in colors; fits are in black. Aluminum 1100 (99% Al) data from [68]. Data for >99.1% Fe (below the Curie point), tungsten, and non-magnetic steel from [2]. Modified from Figure 11.5a,b in the work of Hofmeister [8], with permissions.

Cryogenic data for several metals (Be, Al, Cu, and W) were fitted to Equation (20), which assumes a concave ramp *A* ~ *ν*^2^. The fits ([8]; Figure 11.9 therein) matched the rise to the peak in *K* near 20 to 50 K, but they poorly reproduced the tail. The greater variation in *K* for metals and alloys requires a more complex representation of their absorption spectra. Figure 8 shows that data measured over wide temperature ranges are fitted by summing the formulae for two ramps (four parameters). Using four parameters improves the agreement when the peak is wider, when multiple peaks exist, or when the decline in *K* is clearly not simply due to the single low-*T* peak, as in corundum (Figure 7a).

The examples in Figure 7 and Figure 8 confirm not only that the essential features of *K*(*T*) are reproduced by our analytical model in Section 3, but furthermore that the details can be reproduced using simple representations of absorption spectra.

#### 5.3.2. Thermal Diffusivity Measurements above Ambient Temperature

The thermal diffusivity of diverse insulating and semiconducting crystals with *L* > ~1 mm and *T* > ~280 K is described by

(21) D(T)=FT−g+HT or D(T)=F*298Tg+HT 

where *F*, F*, *g*, and *H* are fitting coefficients, such that *g* is near unity and *H* is small (~0.0002 mm^2^ s^−1^ K^−1^) yet is important at high *T* [60]. The universality of Equation (21) is supported by measurements now encompassing >250 samples, including glasses, melts, ceramics, minerals, rocks, and metals [8].

**Figure 9:**
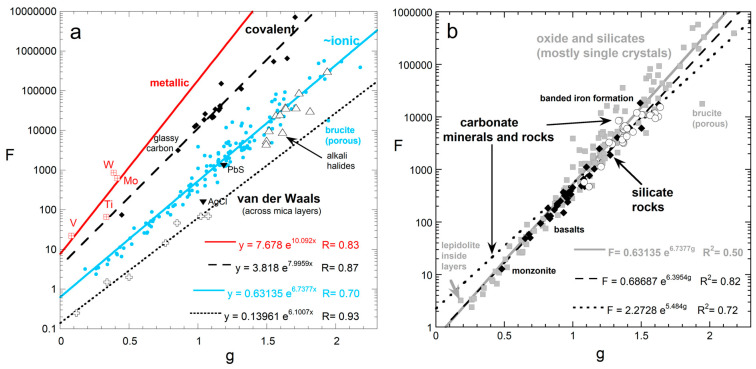
Correlation of fitting coefficients *F* and *g* obtained using Equation (21): (**a**) Metals from [20]; the remaining 173 samples from [8]. Fits for anisotropic substances were made to each orientation. Exponential fits are shown. Porous samples (e.g., brucite, Mg(OH)_2_,) fall below the curves. Salts lie slightly below the curve for the silicates, which have ionic-covalent bonding. (**b**) Comparison of grainy mixtures to crystals. Black diamonds and dashed line = 35 LFA measurements on polycrystalline minerals and rocks compiled by [8]. Square with cross and dotted curve = carbonate minerals and rocks [70]. Grey dots and thick curve = LFA data on oxide and silicate minerals, excluding micas with flow across layers. Part (**b**) was modified from Figure 1 in the work of Merriman et al. [32], with permissions.

The power-law term describes the decline in *K* vs. *T* at moderate temperatures (Figure 7). The parameters *F* and *g* are correlated (Figure 9). Hence, knowing *D* at 298 K (i.e., F*) largely determines *D*(*T*) below ~1000 K. Our radiative diffusion model links the power law in Equation (21) to diffusion of radiation at low frequencies (Section 3), as confirmed in Section 5.3.1.

The linear term (*H* > 0) describes high-temperature behavior including molten metals [20]. For simple structures (e.g., Ge or diatomics), *H* is negligible, but for complex structures (e.g., graphite, silicate glasses, and minerals), *H* is significant [8,60].

Thermal conductivity increasing with *T* has long been linked to the diffusion of radiation in the visible spectral region at high temperatures [44]. The popular form *K* ~ *T*^3^ holds only if the material has constant *A* for all frequencies and temperatures. Using spectral measurements for the mineral olivine ((Mg_0_._9_Fe_0_._1_)_2_SiO_4_) in Equation (17) while using scattering to avoid infinities with *A* = 0 instead provided *K* = a*T* + b*T*^2^ [69].

#### 5.3.3. Pressure Response of Heat Transport Properties

Because any amount of light can fit in any given space, *ℑ* is independent of pressure. Taking the pressure derivative of Fourier’s macroscopic Equation (1), the LHS gives [26,27]

(22) $$\frac{1}{K}{\left.\frac{\partial K}{\partial P}\right|}_{T}=-\frac{1}{V^{1/3}}\frac{\partial V^{1/3}}{\partial P}+\alpha \frac{\partial \alpha^{-1}}{\partial P}\;≅\frac{1/3}{B_{T}}+\frac{7}{B_{T}}$$

which was confirmed by accurate thermal conductivity measurements involving >1 mm samples and *P* < 2 GPa [26].

Taking the *P* derivative of the definition in Equation (11) leads to a relationship for *c_P_* [28], which, when combined with Equation (2), gives

(23) $${\left.\frac{1}{c_{P}}\frac{\partial c_{P}}{\partial P}\right|}_{T}=\frac{-1}{B_{T}}\left\{1-\frac{\Delta Q_{\mathrm{ext}}}{Mc_{P}}\frac{1}{B_{T}}\frac{\partial B_{T}}{\partial T}\right\}≅\frac{-1}{B_{T}},\;\mathrm{hence}\;{\left.\frac{1}{D}\frac{\partial D}{\partial P}\right|}_{T}={\left.\frac{1}{K}\frac{\partial K}{\partial P}\right|}_{T}$$

Available data support Equations (22) and (23) within experimental uncertainty [26,28].

Heat flowing independently through matter underlies the above experimentally confirmed equations. A radiative diffusion mechanism is required.

### 5.4. Effect of Heat Performing P-V Work on Static Properties

Expansion of the solid upon adding heat is measured by many techniques. Interferometry and dilatometry are the most accurate [71], whereas X-ray diffraction data are the most abundant. Diverse comparisons confirm that the strength of the solid limits the amount of *P*-*V* work that can be done when heat is added.

#### 5.4.1. Comparison of the Temperature Responses of Thermal Expansivity and Specific Heat

**Figure 10:**
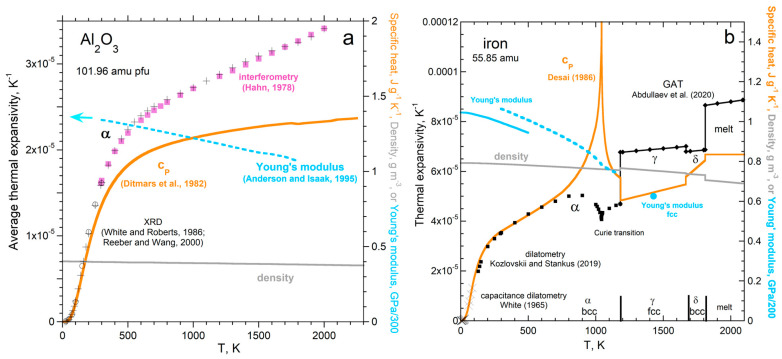
Comparison of thermal expansivity to specific heat: (**a**) Al_2_O_3_. Circle = α from powder XRD compiled by Reeber and Wang [76]; + = α compiled by White and Roberts [77]. Pink squares = single-crystal interferometry and twin telemicroscope measurements of Hahn [78]. Orange curve = *c_P_* compiled by Ditmars et al. [65]. Grey = density calculated from α. (**b**) Fe metal. Thick vertical bars mark structural phase transitions. Orange curve = *c_P_* compiled by Desai [79]; × = capacitance measurements of α-Fe by White [80]. Squares = dilatometry by Kozlovskii and Stankus [81]. Diamonds = gamma-ray attenuation by Abdullaev et al. [82]. Grey = density calculated from α. Part (**a**) was modified from Figure A1a in the work of Hofmeister et al. [28], which has a Creative Commons license.

By averaging datasets on many solids, Bodryakov and colleagues [72,73,74,75] established that α and *c_P_* respond similarly to increasing temperature over a wide *T* range. Comparing highly accurate datasets without phase transitions [28] shows that α(*T*) is proportional to *c_P_*(*T*) at very low *T*, such that the proportionality gradually increases with *T*. At high *T*, the dependence of α on *T* becomes nearly proportional to *T* × *c_P_*. Figure 10a provides the example of corundum. Additional examples (Al, Mo, Ta, Au, diamond, Si, NaCl, KCl, MgO, and Y_3_Al_5_O_12_) are shown graphically in [28] (Appendix A therein). Data on quartz are presented in Section 5.5 on dynamic properties.

Thermal expansivity increases more rapidly with *T* at high *T* than specific heat, because material strength decreases with *T* (Figure 10a). At low *T*, Young’s modulus is nearly constant, so α(*T*) differs from *c_P_*(*T*) only by a numerical constant, per Equation (14). At high *T*, Young’s modulus depends linearly on *T*, so the relationship between α(*T*) and *c_P_*(*T*) is a constant × *T*, as deduced by [72,73,74,75] from averaging datasets. Accurate datasets show that the transition between very-low-*T* behavior and very-high-*T* behavior is gradual. Thus, the pressure–volume work performed in a solid by heat is limited by the material’s strength and its variation with temperature.

The present paper takes the analysis of [28] further by considering phase transitions in detail. Phase transitions generally affect α and *c_P_* in opposite directions, as exemplified in Figure 10b for Fe. Strength decreasing with *T* provides a similar overall increase in α(*T*) compared with *c_P_*(*T*) for Fe, but several phase transitions substantially perturb the *P*-*V* work done by heat.

- As *T* increases, the magnetic Curie transition consumes energy, so raising the temperature by 1 K near 1043 K requires more heat, creating a peak in *c_P_.* Magnetic work is performed, which largely replaces *P*-*V* work. Consequently, the lattice expands less over a given interval of Δ*T* during the Curie transition.
- Iron has a structural transition from 8- to 12-coordination at 1211 K, which increases *ρ* by ~8%. Because the bonds are longer, they are weaker, and so the face centered cubic (fcc) phase has much higher α than the body centered cubic phase (bcc). With the closer packing, less heat is needed to warm the lattice by 1 K, so *c_P_* drops across the bcc-to-fcc transition. Young’s modulus was only measured at one temperature for γ-Fe, but it seemed lower than the extrapolated trend for the α-Fe phase.
- Returning to a bcc structure at 1667 K affects the properties in the opposite direction, which is consistent with our model.
- Melting at 1881 K greatly weakens the structure, so α is large. Lower *ρ* of melts (smaller average bond length) is consistent with higher *c_P_*.

#### 5.4.2. Ambient Temperature Tests

**Figure 11:**
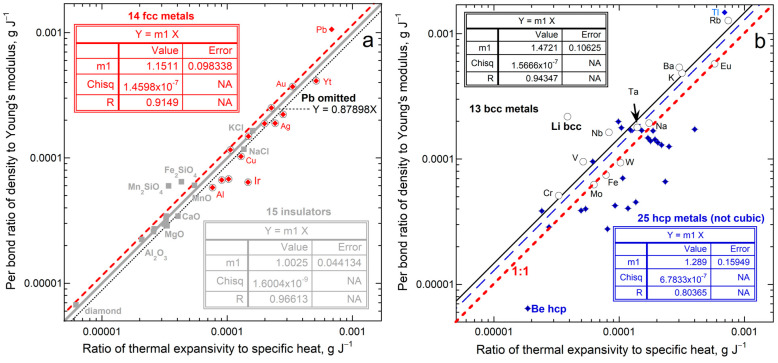
Dependence of α/*c_P_* on *ρ*/(Ξ*N*/*Z*). Data on elements compiled in [28]. For the insulators, tables from [83] were used, omitting Co_2_SiO_4_ because α was estimated. Fits are least squares: (**a**) Insulators and cubic fcc metals. Lead strongly influences the power-law fit. Orthorhombic Fe_2_SiO_4_ has a shearing transition, whereas α for orthorhombic Mn_2_SiO_4_ is unconfirmed. (**b**) Cubic bcc and hexagonal hcp metals. Outliers Li and Be have very small cations and few valance electrons. Both parts reproduced from Figure 14 in the work of Hofmeister et al. [28], which has a Creative Commons license.

**Figure 13:**
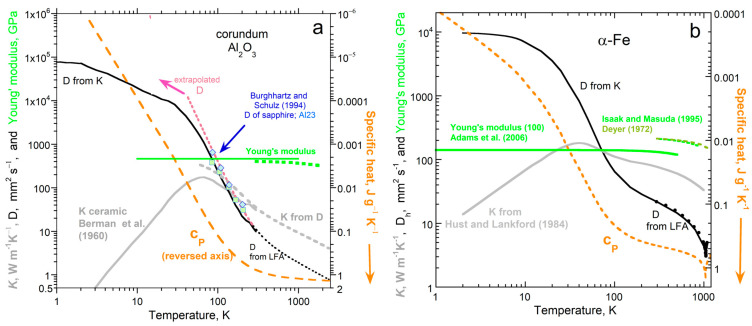
Comparison of transport properties to specific heat on logarithmic axes (with the *c_P_* axis reversed, per Equation (15)), as indicated by the orange arrow: (**a**) Corundum. Dotted line = high-*T D* from Hofmeister [61] extrapolated to low *T* (pink), which is consistent with LFA data on sapphire crystals (blue diamonds from Burghartz and Schulz [93]). The latter study reported a fit to the data, which were scattered. Values of *K* are low due to contact losses; the grey dashed curve suggests intrinsic *K* values. Specific heat (orange curve from Ditmars et al. [65]) increases over all *T* (reversed axis) but is not as complex as the decrease in *D* with *T*. (**b**) Metallic iron in the low-*T* bcc phase. Grey curve shows the highest *K* from [2] to represent the purest sample. *D* calculated using *c_P_* (orange curve from Desai [79]) and density (see Figure 10) is consistent with the Fe standard used in LFA cross-checks, see Henderson et al. [94].

Equations (13) and (14) at ambient temperature were confirmed by the ratio α/*c_P_* depending linearly on the ratio of *ρ*/Ξ for 69 elements and 5 insulators under ambient conditions ([28]; Figures 13 and 14 therein). Figure 11 evaluates Equation (14), which includes coordination numbers. Cubic substances show good agreement of data with the model, consistent with assuming isotropic, spherical forces.

#### 5.4.3. Further Tests on the Effect of Elevated Temperature

**Figure 12:**
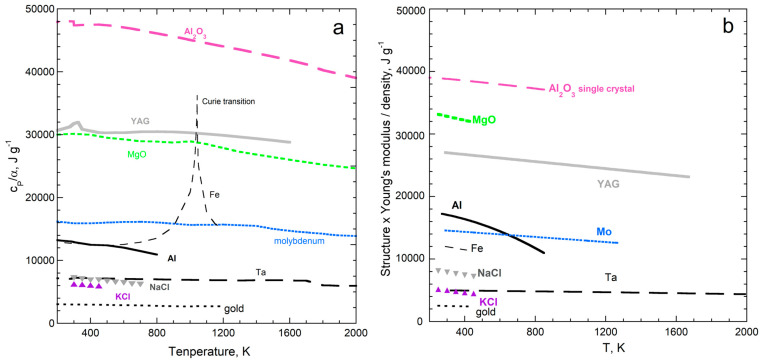
Evaluation of Equation (14) at high *T* for well-studied solids: (**a**) Dependence of *c_P_*_/_α on temperature. See [28] for data sources. Discontinuities in Ta α result from variations between studies. (**b**) Dependence of Ξ/*ρ* with the structural factor on *T*. Constant ambient *ρ* was used, since Young’s modulus is uncertain. Data on Ξ from [84,85,86,87,88,89,90]. For Au, Fe, MgO, NaCl, and KCl, we used *T* derivatives near and above 298 K for *B* and *G* from [91] to compute dΞ/d*T*.

Figure 12 compares *c_P_*/α to (Ξ*N*/*Z*)/*ρ*, based on the units of J g^−1^ representing specific energy. The graphs begin at 200 K, because previous assessments of cryogenic data on *c_P_*_/_α [72,73,74,75] show that this is constant, whereas density varies little, and Young’s modulus is also considered to approach a constant as *T*→0 [84]. Given that the structure factor (*N*/*Z*) is an approximation, the correspondence is good.

### 5.5. The Link of Dynamic to Static Properties

This section explores relationships of *D* (and/or *K*) to only specific heat, because the ties of *c_P_* to other thermodynamic and mechanical properties, as delineated above, can be used to relate *D* (or *K*) to other static properties. For corundum and iron, heat transport properties are accurately measured, so these are the focus. Quartz (SiO_2_) is also examined, because its structural transition is displacive, not reconstructive. Silicate glasses are briefly covered to further ascertain the effect of melting.

Accurate data on these well-studied substances reveal converse behavior of *D* to *c_P_* proposed in Equation (15).

#### 5.5.1. Behavior of a Single Phase

For a single phase, as *T* increases, *c_P_* increases while *D* decreases (Figure 13; note the reverse axis for *c_P_*). Both changes are monotonic. At the limit of absolute zero, *c_P_*→0, whereas *D* is large, finite, and weakly depends on *T*. Although the existence of little heat describes matter near absolute zero per the blackbody curve, the rate of heat transport is fast because negligible heat is stored, so virtually all of the heat added is available to flow. At absolute zero, *K*→0, as shown in many studies [59], which results from unavailability of heat. Because *K* = *Dρc_P_*, *D* must be finite for both *K* and *c_P_* to approach 0 as *T*→0.

The peak in *K* (Figure 13) results from *c_P_* increasing strongly at cryogenic temperatures, but weakly at high *T*, in combination with *D* decreasing slowly at first, and then more rapidly. Because the transitions between the low- and high-*T* regimes are gradual, the peak in *K* is fairly broad. The peak in *K* is unconnected with any change in the mechanism of heat transfer. Instead, as discussed by [92], different signs for ∂*K*/∂*T* originate in macroscopic behavior. The difference is dictated largely by the uptake of heat by the lattice, and by differences in the responses of various material properties to temperature in the low- and high-*T* regimes.

Slight disagreements with Equation (15) for Al_2_O_3_ are connected with differences between the measurements of ceramic and gem-quality samples. For example, *K* from low-*T* LFA data on ceramic Al23 agrees better with the LFA determinations of *K* (grey line [64]), but it lacks the bump near 250 K.

#### 5.5.2. Effects of Magnetic and Reconstructive Phase Transitions on Heat Transport

Metallic iron has many transitions and is important to physical sciences and engineering, which has motivated many experimental studies. All of its structural transitions are reconstructive.

**Figure 14:**
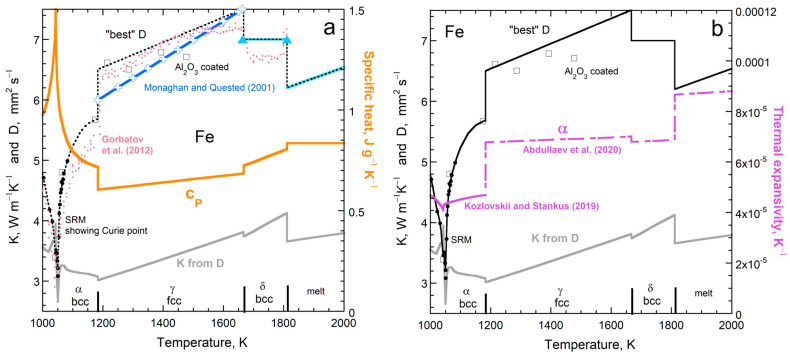
Comparison of transport properties to static properties for metallic iron at high *T*. Axes are linear: (**a**) Specific heat (orange, from [79], which is lower for fcc due to its high density. Black dots show *D* for the Fe standard used in LFA cross-checks from Henderson et al. [94]. Open squares are new data on electrolytic iron coated with Al_2_O_3_ to avoid reaction at high *T*. Blue lines and symbols are LFA data collected with a zirconium coating Monaghan and Quested [95]. Pink dots are plane-wave data from Gorbatov et al. [96], which is a periodic technique; minor contact losses explain the lower values. Dotted line = the best representation of these studies, combined. Grey = *K* calculated from the best estimate. (**b**) Thermal expansivity (pink solid line is dilatometry Kozlovskii and Stankus [81]; dot-dashed line is the gamma attenuation Abdullaev et al. [82]).

Thermal diffusivity changes in the opposite direction to specific heat during each transition, magnetic or structural. The properties in Figure 14a were scaled so that the changes over the Curie transition were similar for *c_P_* and *D*. Changes at the structural transitions are larger (percentage-wise) for *D* than for *c_P_*. Thermal conductivity generally increases with *T* due to both *D* and *c_P_* generally increasing as *T* climbs, whereas the changes in *K* and *c_P_* at the transition vary in diverse ways, since *K* = *Dρc_P_*. The complex pattern in *K* near 1043 K is due to interpolating the various datasets, combined with slight differences in the Curie transition during the various experiments, which is affected by kinetics (heating rate).

Except upon melting, thermal diffusivity responds similarly to temperature and phase changes as does thermal expansivity (Figure 14b). Because liquids are much weaker than solids and are viscous rather than rigid, they expand readily. Direct correspondence of *D* with *c_P_*, but not with α, upon melting emphasizes that transport properties depend on heat uptake, which is independent of strength but depends on the absorbance of light by the material.

#### 5.5.3. The Displacive Transition in Quartz

**Figure 15:**
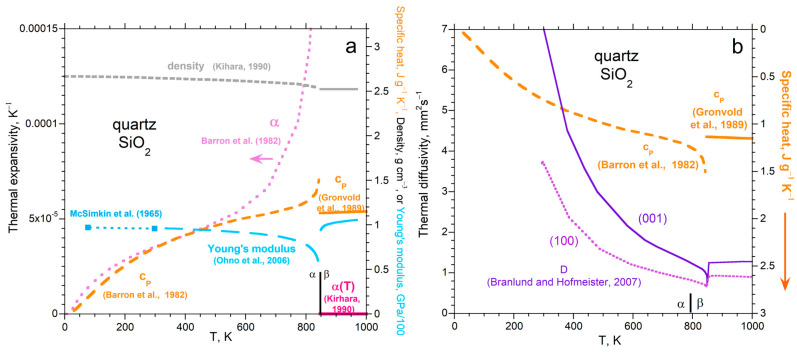
Properties of low- and high-quartz polymorphs: (**a**) Static properties. Data sources are labeled; see McSkimin et al., Barron et al., Grønvold et al., Kihara, and Ohno et al. [97,98,99,100,101]. (**b**) Comparison of thermal diffusivity for nearly pure and anhydrous natural quartz (sample HQ from Branlund and Hofmeister [55]) to specific heat (reversed axis) for a bulk quartz sample from Barron et al. [98] and Grønvold et al. [99].

Quartz consists of corner-linked SiO_4_^4−^ tetrahedra. At low *T*, a trigonal structure (α- or low-quartz) is stable. Transformation to the hexagonal (β- or high-quartz) structure at 846 K involves rotation of the tetrahedra with respect to each other and provides lower density (Figure 15a). No bonds are broken in this displacive transition.

Thermal expansivity, specific heat, and Young’s modulus change much more abruptly than *ρ*. The relationships among the properties (Figure 15a) differ from those of the reconstructive transitions in iron (Figure 14). The silica tetrahedra themselves are little affected by changes in *T* or *P*, so the measured physical properties reflect changes in the tilting and rotation of the tetrahedra. After the displacive transition, β-SiO_2_ is stronger than α-SiO_2_ even if the latter is at absolute zero. The density of β is constant within experimental uncertainty, consistent with its strength. However, changes in specific heat are minor, confirming that these properties are controlled by different factors.

Thermal diffusivity for quartz (Figure 15b) changes with *T* in the opposite direction to *c_P_*, similar to Figure 13 for single phases and Figure 14 for reconstructive transitions. Because all other physical properties respond differently than *c_P_* to the transition (Figure 15a), the behavior of quartz confirms that the rate of heat transport is regulated by heat uptake, rather than by the equation of state or elastic properties.

#### 5.5.4. Melting of Silicate Glasses

**Figure 16:**
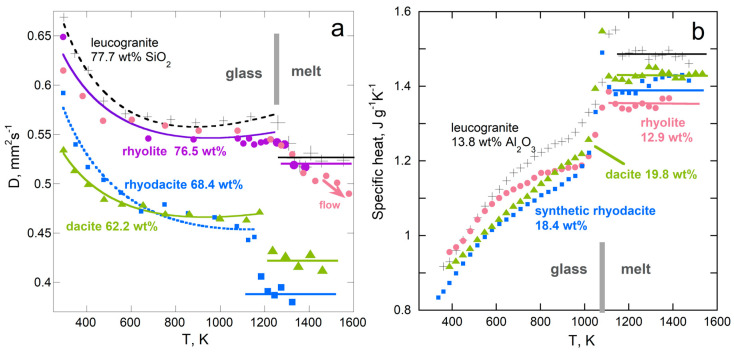
Physical properties of glasses and melts made by remelting natural lavas with high silica contents: (**a**) Thermal diffusivity. Silica content is labeled. Pink and purple dots are two slightly different glasses from [104]. (**b**) Specific heat from powders of the identical glasses. The alumina content is labeled. Samples have <5 wt% each of oxides of Fe, Ca, Mg, Na, and K. Modified from the work of Hofmeister et al. [102], Figures 3a and 4a therein, with permissions.

**Figure 17:**
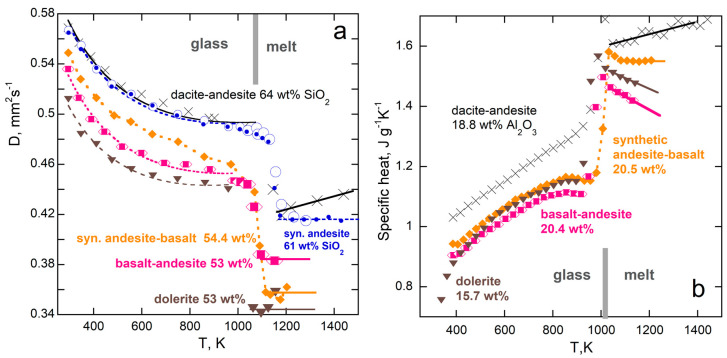
Physical properties of glasses and melts made by remelting natural lavas with intermediate silica contents: (**a**) Thermal diffusivity. Silica content is labeled. Blue dots are a synthetic andesite with trace fluorine. (**b**) Specific heat from powders of the identical glasses, except for the andesites. Alumina content is labeled. Samples have <10 wt% each of oxides of Fe, Ca, and Mg, and <5 wt% oxides of Na and K. Modified from the work of Hofmeister et al. [102], Figures 3b and 4b therein, with permission.

Thermal diffusivity is compared to the specific heat of various silicate glasses in Figure 16 and Figure 17. Results from [102] are presented because a wide compositional range was probed and the same samples were used in various experiments, although larger pieces were used to measure *D* than to measure *c_P_*. The behavior is similar for other silicate glasses [103].

High-silica samples have high viscosity in both glass and melted states. Upon melting, the samples retain their shape, but as the temperature is further raised, samples can either flow, or outgas, or crystallize. Melts of the stiffest samples have constant *c_P_* and nearly constant *D* (Figure 16). For all samples studied, *D* decreases upon melting, whereas *c_P_* increases.

For less-viscous, low-silica samples, *D* also decreases upon melting, whereas *c_P_* increases (Figure 17). The changes are larger than for the high-silica samples, but the behavior after melting is less regular, as these lower-viscosity samples are prone to flow, which permits outgassing of trace water dissolved. The concentrations of the divalent ions are larger, particularly for Fe, which has multiple valence states. Hence, the absorption spectra are more varied in the near-IR–visible range, as shown in [102], and so are the responses of low-silica glasses to adding heat.

Thus, the static and dynamic properties of electrical insulators and iron metal both respond similarly to melting, and both are in accord with Equation (15).

## 6. Discussion and Conclusions

### 6.1. A Novel Approach

Because heat and mass diffuse independently in solids but together in gas, models based on the kinetic theory of gas (e.g., phonon and electron scattering) inadequately describe the heat transport properties of solids (Appendix A). To better understand heat transport in solids and its relationship with stored heat, this paper, as in our previous efforts, combines a theoretical analysis that focuses on the unique attributes of solids with accurate laser flash analysis data on their heat transport properties.

#### 6.1.1. Theoretical Component

Our macroscopic approach focuses on Fourier’s equations. These laws do not depend on any particular microscopic mechanism, yet they require that heat flows through condensed matter independent of the motions of its constituent atoms (Figure 1a and Figure 2a). Augmentation of stored heat is essential to raise the temperature. Thus, how much heat is taken up by matter during the application of external heat (i.e., how much heat is removed from the flow) is central to understanding heat transport. Macroscopic definitions of specific heat describe the uptake of added heat by matter, along with its consequent increase in temperature, as discussed in detail here. The added heat also performs pressure–volume work, thereby expanding the solid, and so the static properties of thermal expansivity, which is the manifestation of *P*-*V* work done, are also germane along with Young’s modulus (i.e., rigidity or strength), which quantifies the resistance of a material to *P*-*V* work.

Our theoretical approach is innovative because rigidity is included in our discussions of static properties (reviewed here) and dynamic transport properties (introduced here). Neither rigidity nor shear moduli were incorporated in prior thermodynamic models.

Dimensional analysis and differentiation provide testable equations (Section 2). Based on the deduced length-scale dependence and Fourier’s assumption that heat flows through a solid, the microscopic component of our approach evaluates the integral equation for radiative diffusion that has been widely used to probe diffusion of visible light. We use idealized infrared absorption spectra (Section 3). These formulae also are testable.

#### 6.1.2. Experimental Component

Testing (Section 5) focused on well-studied solids, including corundum, quartz, pure Fe metal, and graphite, which together span the main bonding types. Other crystalline metals and alloys were also included. Some highly viscous silicate melts are also discussed, as these are viscous rather than rigid [105].

### 6.2. Key Findings

#### 6.2.1. Review of Our Previous Work

The present paper emphasizes key findings from our previous efforts on how temperature affects static and dynamic properties:

- Thermal diffusivity depends linearly on length for thin metallic, semiconducting, and electrically insulating solids, but it is constant when *L* exceeds ~2 mm (a condition that is commonly explored in the laboratory). A medium is required for diffusion to occur.
- A radiative diffusion model reproduces measured thermal conductivity (*K* = *Dρc_P_*) for thick metals and insulators from ~0 to >1200 K using idealized spectra represented by 2–4 parameters.
- Heat conduction consists of diffusion of light at low frequencies of the infrared region.
- Because added heat performs *P*-*V* work, thermal expansivity is proportional to *ρc_P_*/Young’s modulus (i.e., rigidity or strength).
- For completeness, Section 5.3.3 summarizes our identity for the pressure dependence of *K* (extracted from Fourier’s law), and of specific heat (from considering steady-state conditions), and uses these to provide ∂*D*/∂*P*. For validation, see [26].
- The present paper further develops two earlier findings, summarized as follows:
- During phase transitions, α can be affected differently than *c_P_*, depending on how the associated changes alter the uptake of heat, structure, and rigidity of the material.
- The peak in *K*(*T*) in the cryogenic regime results from opposite responses of *c_P_* and *D* as *T* increases. Specifically, *c_P_* increases with *T*, whereas *D* is finite, large, and flat near 0 K; this trend then merges with a power-law decline describing conditions near ambient. Little heat is available to move at very low *T*, but it diffuses quickly.

#### 6.2.2. Original Findings

The present paper postulates and confirms the following:

- Greater uptake of applied heat (e.g., *c_P_* generally increasing with *T* or at certain phase transitions) reduces the amount of heat that can flow through the solid, but because *K* = *Dρc_P_*, the rate (i.e., *D*) must substantially decrease.
- Consequently, as *T* changes, thermal diffusivity responds in the opposite direction to changes in *c_P_*.

Some discussion is warranted.

### 6.3. Why Static and Dynamic Properties Are Linked

The idealization of an isothermal state, which is a key reference point in classical thermodynamics, is difficult to achieve because heat always flows. No material is either a perfect thermal insulator or a perfect thermal conductor. However, sequential isothermal segments are achieved under steady-state conditions (Figure 2a). Adopting steady-state conditions as the key reference point revealed links between static and dynamic properties. This link is exemplified by the Stefan–Boltzmann law relating temperature to flux, a key parameter in Fourier’s model, which depicts the time rate at which heat is applied.

Measurements of thermodynamic properties are carried out slowly in order to maintain equilibrium at each step. Determinations of transport properties are also impacted by the heating rate, as is evident from the known difficulties in reaching a steady state in thermal conductivity experiments [11]. This difficulty is addressed by laser flash analysis, as this technique infers *D* during the approach of a material to equilibrium after a small increase in heat is added (Figure 1).

Dynamic and static properties are linked partially because straying appreciably from near-equilibrium conditions is minimized in experiments, and partially because the key thermodynamic property of specific heat describes heat uptake and storage, both of which are related to the interaction of light with the material. The infrared region is most important for both storage and transport at common laboratory temperatures. Approximations of infrared spectra in extensions of Debye’s and Einstein’s models reproduce heat capacity vs. *T* for simple and complex materials [46,47,48]. Approximate spectra suffice in summations (giving *c_P_*) or integration (giving *K*, Equation (17)), because both are smoothing operations.

### 6.4. How Static and Dynamic Properties Differ

Heat (pure energy) is a dynamic entity. Although a material stores energy in its lattice as vibrational modes, this storage is replenished by flux from the surroundings. Otherwise, the sample cools. Although *c_P_* and *D* are both measured through perturbing the system by adding heat, the static approach probes the stable state, whereas the dynamic approach probes the time dependence of changing from one state to another. Differences between static and dynamic conditions have the following consequences:

- Specific heat is a material property, whereas *D* and *K* depend on the length across which heat flows under certain circumstances. The length dependence results when *L* is sufficiently small that the sample partially transmits (i.e., negligibly absorbs) much of the blackbody radiation (ballistic heat transfer). The change from diffusive (absorbing) to ballistic (transmitting) conditions is continuous, so *D* depends linearly on *L* at small *L*.
- Added heat that is absorbed (taken up) both increases *T* and performs work, and so thermal expansivity depends directly on both *c_P_* and Young’s modulus. In contrast, thermal diffusivity responds oppositely to the changes in *c_P_* as *T* increases, as *D* describes heat that is moving, not stored.

### 6.5. Mechanisms for Heat Storage and Transport, with Implications

Although a macroscopic model (e.g., Section 2) cannot distinguish between microscopic mechanisms, clues exist in the experimental results. An improved understanding of the processes of externally added heat performing work and excess added heat flowing through the solid helps to infer microscopic mechanisms, as follows:

#### 6.5.1. Storage Mechanisms

The above focuses on energy locked into vibrations of materials and stored in elastic properties of solids. Storage mechanisms involving electronic or magnetic processes are probed using independent electrical or magnetic measurements, as discussed in many textbooks.

Another type of energy storage is latent heat, which is the heat that must be added to transform a solid to a liquid without raising its temperature. Based on the validated Equation (14) plus Figure 14b, Figure 16, and Figure 17, latent heat equals the change in elastic energy due to reduced rigidity (*G* becomes negligible upon melting). This study further proposes that changes in configurational entropy used to describe the melting of glasses are instead changes in elastic energy. Both ideas are testable.

#### 6.5.2. Transport Mechanisms

The length dependence of *D* and our microscopic model reproducing measurements show that diffusion of radiation at low, relevant infrared frequencies is the main mechanism for heat transport (conduction) in solids, including metals. For transparent materials, boundary-to-boundary transport is important over the short distances in laboratory experiments at certain frequencies, which has caused misunderstandings. For metals, electronic transport is transient, since electrons are fast but carry negligible heat. The concept of electron scattering remains in part because the correct and relevant equations for parallel heat flow were only recently derived [4]. Weak, temporary electronic transport in metals is initially evident in the time–temperature curves of LFA (Figure 1c). The slow, strong rises describe lattice transport, and can also be used to measure heat capacity.

The notion that elastically scattered phonons transport heat in electrical insulators likewise persists, due to the historical errors in thermodynamics of neglecting Fourier’s contribution and overlooking the effect of rigidity on a material. This paper and our previous work incorporate heat transfer and elastic processes into thermodynamics, leading to revisions in classical formulae (Section 2 and Section 5.3.3) and concepts, as summarized in the present section.

#### 6.5.3. Inelastic Interactions

Although the elastic approximation is the starting point for understanding many behaviors of matter, and is used here along with incrementally conserving heat energy, the dissipative nature of heat transport cannot be ignored. We have focused on steady-state behavior, as this situation is piecewise isothermal and removes the effect of time. Exploring steady-state conditions permitted us to deduce and verify how static and dynamic properties are similar and where they differ, and to decipher links between specific heat, thermal expansivity, Young’s modulus, thermal diffusivity, thermal conductivity, and length-scale. The temperature and pressure responses of *D* and *K* are revealed by our experimental verification of our macroscopic equations, by utilizing the theorem of Equation (15), and in our use of approximate spectra in our radiative diffusion model.

### 6.6. Implications for Applied Sciences

Ideas developed and validated in the present paper and in [28] pertain to thermal and thermo-structural analysis and design in a wide range of industries. Equations (14) and (15) combine physical properties that are used in structural and thermal models in engineering. Ranges in parameters are limiting factors in the design of materials and systems, and so our formulae are potentially important for practical matters.

The interiors of planets and stars are of great interest to the geologic, planetary, and astronomical sciences. However, the extreme conditions of simultaneously high *T* and *P* are difficult or impossible to achieve in static laboratory experiments. Our model should help with modeling processes inside planets and stars that involve both deformation and heat transport over immense scales of time and distance.

## Figures and Tables

**Figure A1:**
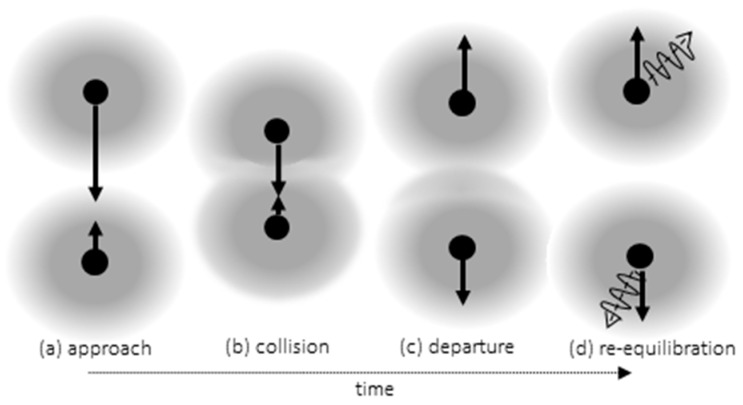
Schematic of dynamic, inelastic interactions of finite-size atoms in a monatomic gas. Left to right shows the progression of an inelastic collision with time. Black = nuclei. Grey = shells of electrons, forming a shielding cloud. Squiggle arrow = thermal photon. (**a**) Head-on approach of two spherical atoms with different speeds. (**b**) Collision, where a reduction in distance along the one direction increases the Coulombic repulsion and breaks the spherical symmetry, which changes the energy of the atom. The energy needed to deform the atom is obtained from the kinetic energy reservoir. (**c**) Departure, where the atoms back-transform to a spherical shape with lower energy. (**d**) After the interaction ceases, the atoms must shed the energy difference between the excited and original states as heat: Consequently, the total energy of translation is reduced. Adapted from the work of Hofmeister [8] (Figure 5.2 therein), with permissions.

## Data Availability

Virtually all data used in this paper are previously published. The few new measurements are described in detail here.

## Appendix A

Problems in the currently popular phonon and electron gas models for heat transport in solids can be traced to the kinetic theory of gas (KTG). Our discussion below focuses on the common, Cartesian geometry in laboratory experiments, i.e., Equation (1) pertains.

### Appendix A.1. Limitations of the Kinetic Theory of Gas

In the KTG, temperature is linked to the random motions of molecules in all three Cartesian directions. This assumption is reasonable for static properties, e.g., heat capacity. But, considering the three directions to be equivalent provides the factor of ⅓ in the well-known formula, as stated in many books, e.g., [106] However, heat flows down the thermal gradient, per the left-hand side (LHS) of Equation (1). Thus, only one direction is relevant. This finding is corroborated by examining the formulation: KTG casts the thermal conductivity of a single molecule (*K_i_*) as the product of its heat capacity per molecule (*c_i_*) with its speed (*u_i_*) and the distance between collisions (*λ_i_*), and then averages (angle brackets) the summation over *N* molecules in the relevant volume and over the three directions. Hence,

(A1) $$K_{gas}=\frac{\sum u_{i}c_{i}\lambda_{i}}{3N}=\frac{\sum K_{i}}{3N}=\frac{\langle K_{i}\rangle }{3}$$

However, the RHS is erroneous because

(A2) $$\;\frac{\langle K_{i}\rangle }{3}\equiv \frac{K}{3}\neq \;K$$

Independent confirmation is provided by [4].

Although the mean free path between collisions is relevant to momentum exchange, this does not control the value of *K*, as follows per [8] (p. 158ff). Lifetimes (*τ*) sum inversely by definition [106]; thus, so do the mean free paths. Thus, the shortest lifetime (or *λ*) controls *K_i_* and the average *K*. The time during the collision (interaction time) is much shorter than the time between collisions. Hence, the mean free path between collisions is not relevant. What happens during the collisions is extremely important.

Atoms or molecules are not hard spheres and will deform or shear; these events classically generate heat (Figure A1). Collisions, being inelastic, make the transport properties dynamic, not static.

### Appendix A.2. Repercussions of Microscopic Mechanisms for Solids

The above flaws in the KTG are carried over to phonon scattering models for insulators and electron scattering models for metals. Foremost, these are elastic models based on scattering.

Regarding metals, Sommerfeld’s Lorenz number in the Wiedemann–Franz (WF) law includes the problematic factor of ⅓. Agreement with data on metals and alloys is fortuitous, in addition to agreement only holding approximately near ambient temperature [4], see Figure 9.1, p. 298 in [20]). Many have pointed out the failures of the WF law, e.g., [2]. For detailed discussion of additional serious problems associated with a dominantly electronic mechanism, including violations of the second law of thermodynamics, see [4,20].

Regarding insulators, several problems exist; see [8] (p. 371). First, acoustic modes are generally considered to dominate the summations similar to the above (i.e., Σ*u_i_c_i_λ_i_*), due to their large velocities (~10 km s^−1^), whereas optic modes have low, near-zero velocities near the Brillouin zone center. However, exchange of energy between acoustic and optic modes is unavoidable. Moreover, shear modes do not change the lattice volume (e.g., [107]) and, thus, do not participate in heat transfer [4] other than by secondary coupling. Second, the terms in the summations used have not been weighted for heat capacity, which violates the conservation of energy. Because optic modes carry most of the heat in a solid, these will dominate a weighted sum. Third, acoustic waves propogate whether or not heat is applied, and so they describe a phenomenon other than heat transfer. Moreover, acoustic modes are not strongly attenuated; for example, seismic waves travel 1000s of km, which implies a ballistic response. However, similar rocks are used as hot plates to inhibit the transmittal of heat beyond ~cm distances. Heat is extremely damped compared to sound waves.

The ubiquitous existence of a peak in *K*(*T*) near ~200 K for insulators and metals is relevant. The phonon scattering model postulates that different scattering mechanisms operate at low and high temperatures, where *K* respectively increases and decreases with *T*. Instead, one mechanism for heat transport is expected, because one mechanism provides the lattice heat capacity. These two scattering mechanisms are described as umklapp and normal processes, which refer to the directions of the phonon scattering vectors. Energy flow is reversed by umklapp events (e.g., [47]). This flow reversal is inconsistent with the LHS of Equation (1).

## Appendix B

### Appendix B.1. Summary of Conventional Methods in Heat Transfer Studies

For detailed discussion of the diverse contact methods applied to many materials, see, e.g., [8,11,14,108,109]. All methods discussed in this subsection record thickness.

For metals and alloys, absolute, conventional, contact methods routinely achieve ±5% accuracy because metals make good physical contact with thermocouples and are opaque at the thicknesses used. Hence, metals are used as standards or for calibration.

In contrast, electrical insulator and semiconductor measurements are affected by thermal losses at physical contacts and opposing gains from ballistic radiation. Reported uncertainties of ±5% for non-metals in conventional studies are nominal because such claims rest on using metal standards. Instead, reduction in *K* by ~10% per physical contact is indicated by measurements that vary the number of contacts used, as well as by theoretical analysis. Augmentation through unwanted ballistic radiative transfer depends on the material’s absorption spectrum (e.g., [51,55]) and whether the technique is steady-state or periodic.

For steady-state methods, spurious ballistic radiation increases with *T* or even more strongly, becoming overwhelmingly large by ~700 K; such contact experiments are limited to ~1000 K.

Augmentation is lower for periodic methods, where behavior during a temperature excursion is compared to a mean *T* [108] but is revealed by comparisons. The “transient” divided bar method [110] is actually periodic, along with Angstrom’s (e.g., [111]) and the “transient” plane source methods [112]. Periodic methods do not resolve behavior as a function of time, and so they assume that *K* or *D* is constant over each temperature cycle.

### Appendix B.2. Summary of Modern Methods in Heat Transfer Studies

Two periodic methods are currently popular; see reviews by [12,59,113]. Both have limitations similar to that of Angstrom’s historic method, and in addition neither measures the distance over which heat is transferred. Because this essential constraint is missing, many assumptions are made. These assumptions are cross-checked against previous data on macroscopic samples. However, many applications (e.g., thermoreflectance) probe thin films and epitaxial layers, for which low thicknesses yield lower *D* or *K* (Section 2.1 and Section 5.1).

The 3ω method [114] is based solely on the response of a heating wire deposited on a substrate to periodic changes in the temperature of the surroundings. The results depend on a thermal model of the wire and its substrate (the sample). Ballistic radiation exists inside the sample, but because low *T* is accessed (<400 K), this contribution is small, as confirmed by comparisons.

Thermoreflectance measurements of thin films and layers on substrates [115,116] are not simple [12]. The experiments involve superimposed pulses and layers, for which several properties pertain: density, heat capacity, and thermal diffusivity combine to provide thermal effusivity. In addition, the thermal resistance of the interfaces has an effect, and the heat flow is three-dimensional. Thus, modeling is central to extracting multiple properties [12]. Results are benchmarked against bulk samples, which is a concern. Studies of mica across the layers give reasonable values [117], because bulk (LFA) measurements likewise use thin, cleaved sheets [56].

## Appendix C

New samples in Figure 3b include Poco graphite [63] cut from an LFA standard supplied by Netzsch. X-ray diffraction measurements from the surface showed additional graphene oxide. Foils of Fe, Fe_834_Cr_13_, and stainless steel 304 were purchased on eBay. Electron microprobe analysis confirmed their chemical compositions. Thick stainless steel 18-8, purchased from McMaster-Carr, Chicago, IL, USA, has a higher Ni content and minor Mo but otherwise has a similar chemical composition to our samples, which have Fe~72 atomic % Cr~19%, Ni~6%, Mn~1%, and Si < 1%, with trace Al and a few other elements. Thick steel 304 and Al1100 (with >99.9% purity) were also purchased from McMaster-Carr.

Electrolytic iron, shown in Figure 14b, is 99.97+% purity (metals basis), purchased from Alfa/Aesar, Ward Hill, MA, USA.
